# Distinct Characteristics in Japanese Dermatitis Herpetiformis: A Review of All 91 Japanese Patients over the Last 35 Years

**DOI:** 10.1155/2012/562168

**Published:** 2012-06-12

**Authors:** Chika Ohata, Norito Ishii, Takahiro Hamada, Yutaka Shimomura, Hironori Niizeki, Teruki Dainichi, Minao Furumura, Daisuke Tsuruta, Takashi Hashimoto

**Affiliations:** ^1^Department of Dermatology, Kurume University School of Medicine, 67 Asahimachi, Kurume, Fukuoka 830-0011, Japan; ^2^Kurume University Institute of Cutaneous Cell Biology, 67 Asahimachi, Kurume, Fukuoka 830-0011, Japan; ^3^Laboratory of Genetic Skin Diseases, Niigata University Graduate School of Medical and Dental Sciences, 1-757 Asahimachi-dori, Chuo-ku, Niigata 951-8510, Japan; ^4^Department of Dermatology, National Center for Child Health and Development, 2-10-1 Okura, Setagaya-ku, Tokyo 157-8535, Japan

## Abstract

We reviewed all 91 Japanese dermatitis herpetiformis (DH) patients reported over the last 35 years. The male-to-female ratio was 2 : 1. The mean age at onset was 43.8, and 13 years earlier for female patients. More than half of these Japanese DH patients showed granular IgA deposition in the papillary dermis, and another one-third showed fibrillar IgA deposition. The male patients with granular IgA deposition were 10 years older than those with fibrillar deposition. Whereas patients with granular IgA deposition showed typical distribution of the skin lesions, the predilection sites of DH tended to be spared in patients with fibrillar IgA deposition. Only 3 patients had definite gluten-sensitive enteropathy. There was a statistical difference in the frequency of human leukocyte antigen (HLA)-DR9 between the granular group and controls among Japanese. No patients had HLA-DQ2 or -DQ8, which is frequently found in Caucasian DH patients. The absence of HLA-DQ2/DQ8, the inability to identify celiac disease in most cases, the predominance of fibrillar IgA, and the unusual distribution of clinical lesions in Japanese patients suggest that Japanese DH may be a subset of DH patients and have a pathogenesis which is different from that currently proposed in Caucasian DH patients.

## 1. Introduction

Dermatitis herpetiformis (DH) is a rare, intensely pruritic, chronic and recurrent papulovesicular disease, in which the lesions usually develop symmetrically on the extensor surfaces. This disease can be clearly distinguished from other subepidermal blistering diseases by histopathological and immunological criteria. Biopsy of an early lesion shows collections of neutrophils at the papillary tips, and direct immunofluorescence (DIF) reveals nonlinear (mostly granular, or fibrillar) IgA deposition in the papillary dermis.

DH is most prevalent among the Caucasian population, and several population-based studies have been conducted, which disclosed a close association with gluten-sensitive enteropathy (GSE) and the human leukocyte antigen (HLA)-DQ2 or HLA-DQ8 [1–5]. In contrast, only case reports and one review article have been published in Japan, reflecting rare occurrence of DH in Japan [6–85]. The previous review of Japanese DH cases revealed differences from Caucasian DH, such as a high frequency of fibrillar IgA deposition in the papillary dermis, a rarity of GSE, and the absence of HLA-B8/DR3/DQ2 haplotype [59].

The fibrillar immunofluorescence pattern of IgA deposition in DH was hypothesized to be related to longitudinal sectioning of affected dermal microfibril bundles, while the granular pattern represents transverse sectioning. However, confocal laser-scanning microscopy revealed numerous fibrils stained with anti-IgA antiserum, extending from the dermoepidermal junction to 50 to 110 *μ*m deep in the dermis. They crossed each other at various angles to form a three-dimensional network. Moreover, immune electron microscopy demonstrated the diffuse dispersion of immune deposits on the surface of microfibrils of dermal microfibril bundles [86]. These findings signify that fibrillar IgA deposition is a distinct pattern. Although fibrillar IgA deposition in DH is ignored in some review articles of DH [87–89], it cannot be dismissed if DH is to be understood sufficiently.

To disclose the unique features of Japanese DH, we have reviewed all reports of Japanese DH patients from 1976 to 2011, most of which were written in Japanese [6–85]. We also compare the characteristics of patients with granular IgA deposition to those with fibrillar IgA deposition.

## 2. Materials and Methods

First we selected Japanese DH cases by searching Ichushi Web (ver. 5), a Japanese medical literature database provided by NPO Japan Medical Abstracts Society, using the term, “dermatitis herpetiformis Duhring” in Japanese, and PubMed using the term, “dermatitis herpetiformis AND Japanese.” Then, we also collected all articles for Japanese DH cited by these articles. Eventually, more than 200 articles were collected. Since earlier Japanese reports of DH included linear IgA bullous dermatosis cases, we omitted these cases. Thus, we selected only cases, which showed subepidermal blisters, neutrophilic microabscesses, and nonlinear IgA deposition in the papillary dermis. Finally, 91 Japanese DH cases reported from 1976 to 2011 were accumulated [6–85].

 Because one of the characteristics of Japanese DH is a high frequency of fibrillar IgA deposition, we compared the cases with granular IgA deposition (granular group) and those with fibrillar IgA deposition (fibrillar group). We performed Student's *t*-test for comparison of age distribution, and the *χ* ^2^ test for the HLA study using the SPSS software (ver. 19). A *P* value of less than 0.05 was considered to indicate statistical significance. *P* values for the HLA study were corrected by multiplying the *P* value by the number of antigens tested (HLA-DR = 10).

## 3. Results

### 3.1. Overview of Japanese DH (Table 1)

Ninety-one Japanese DH patients consisted of 61 males aged between 1 and 87 years (mean 51.5 years, SD 20.5) and 30 females aged between 18 and 72 years (mean 36.8 years, SD 14.1). The data on the age at onset of DH were available for 48 males (1–87 years, mean 48.5 years, SD 19.6) and 27 females (14–72 years, mean 35.3 years, SD 13.0). The female patients started suffering from DH 13 years earlier than the male patients. No patients had any family history of DH or celiac disease (CD).

Clinical manifestation was polymorphic, consisting of erythemas, urticarial plaques, papules, and herpetiform vesicles and blisters. Superficial erosions and excoriation due to scratching were also frequently noted. Most patients presented intense pruritus, being mild in other patients. More than half Japanese DH patients had lesions on the predilection sites as in Caucasian DH, that is, the elbow, buttock, knee, face, ear, neck, scalp, and groin. In particular, 44% of Japanese DH patients had lesions on the elbow, buttock, and/or knee. The face, ear, neck, scalp, and groin were affected in only a few patients. Interestingly, 41 and 55 Japanese DH patients presented skin lesions on nonpredilection sites such as the extremities and trunk, respectively, with or without concurrent lesions on predilection sites. Six patients had lesions on the whole body. No mucosal involvement was reported.

Most biopsy specimens showed subepidermal blisters and an accumulation of neutrophils with or without a few eosinophils at the papillary tips. In DIF, 50 (54.9%) cases showed granular IgA deposition (referred as granular group), and 33 (36.3%) cases showed fibrillar IgA deposition in the papillary dermis (referred as fibrillar group). Seven cases showed both granular and fibrillar IgA depositions, and only one case showed cluster IgA deposition [80]. Twenty (22.0%) cases showed C3 deposition, and 9 (9.9%) cases showed IgG deposition in the papillary dermis. No circulating antibodies to the basement membrane zone were shown in the cases for whom indirect immunofluorescence (IIF) results were available.

Gluten-sensitive enteropathy (GSE) was associated with only 3 cases, who responded to gluten-free diet (GFD) with dapsone [24, 47, 59]. However, GFD for one case was not strict, and no information about long-term strict GFD was obtained for another 2 cases. While jejunum biopsy revealed villous atrophy in 3 patients including 1 patient with GSE [14, 39, 59], other 3 patients with no clinical symptoms of gluten sensitivity did not show any change [24, 33, 52].

 Eight cases had diabetes mellitus (DM). One of those had noninsulin-dependent type DM, and four also seemed to have noninsulin-dependent type DM according to their therapy. The type of DM of three cases was unknown. Three cases had lymphoma. One case each had mycosis fungoides and anaplastic large cell lymphoma although the type of lymphoma was unknown in one case [47, 52]. Thyroid disease and Sjögren syndrome were also found in one each case of Japanese DH [9, 59].

The most common HLA antigen found in Japanese DH was Cw3, followed by A2, DR9, A24, and DR4 in the descending order. Compared with the controls Japanese population [90], there was no increase in the frequencies of HLA class I antigens (A, B, and C antigens), whereas there was a slightly increased frequency of HLA-DR9 in all the DH patients examined for HLA. No patient had either HLA DQ2 or DQ8.

Antireticulin, antigliadin and antiendomysial antibodies were investigated in small number of Japanese DH cases, and none had these antibodies. IgA antitransglutaminase antibodies have been reported in only 2 Japanese DH patients. In both cases, antiepidermal transglutaminase (eTG) antibodies were detected, while antitissue transglutaminase (tTG) antibodies were not [84].

Dapsone was effective for most patients. Although most patients treated with dapsone required reduced dosage of dapsone for maintenance therapy, the lesions of 5 patients were completely cleared, and no therapy was required after several-month administration of dapsone without any other treatment [41, 44, 46, 64, 78]. The efficacy of GFD was difficult to evaluate, particularly, in patients without clinical symptoms of GSE because they had dapsone administration concurrently, and GFD was not strict at all [13, 19, 20]. In contrast, GFD seemed to relieve abdominal symptoms in the patients with GSE although GFD for one patient was not strict [24, 47, 59]. Topical steroid was sufficient to cure the lesions completely in some patients. In one case the lesions disappeared 4 months after tonsillectomy [51]. Except for the 3 patients with GSE, no patients developed clinical symptoms of gluten sensitivity throughout the course although they were taking a normal diet.

### 3.2. Comparison of Granular and Fibrillar Groups (Table 2)

The number of cases in the granular group was approximately 1.5 times higher than that in the fibrillar group. In both groups, the male patients were twice the number of the female patients. The mean age at onset of male patients in the granular group was almost 10 years older than that in the fibrillar group although no statistical significance was obtained. The mean ages at onset of female patients in both groups were relatively close. The mean ages at onset of the male and female patients were relatively close in the fibrillar group, while the mean age at onset of the male patients was 15 years older than that of the female patients in the granular group.

 Patients in the granular group had lesions on the elbow, knee, buttock, face, ear, neck, scalp, and/or groin, which were common sites in the Caucasian DH patients, more frequently than those in the fibrillar group. Particularly, patients in the granular group had lesions on the elbow, knee, and/or buttock almost three times as frequently as those in the fibrillar group. C3 deposition was more frequently seen in the granular group than the fibrillar group. Comparison for small bowel disease and associated diseases was difficult because of a small number of cases involving these diseases in both groups. Although there were no statistical differences in the frequency of the HLA type between the granular and the fibrillar groups, a statistical difference in the frequency of HLA-DR9 between the granular group and the controls was found (corrected *P* = 0.02).

## 4. Discussion

The mean age of Japanese DH patients at the initial visit was 46.6, with a male predominance of 2 : 1. The age range and the male-to-female ratio in Japanese DH study are very similar to those in the Caucasian study [5, 91, 92]. Caucasian female patients with DH also tend to develop skin lesions at a younger age than male patients [5, 92]. Although a high incidence of familial DH has been reported in Caucasian patients [93, 94], no family history was found in Japanese patients. The clinical manifestation and distribution, as well as histopathological features, were also similar to those found in Caucasians [95, 96].

The distinct results of DIF in Japanese DH were noteworthy. More than one-third (36.3%) of Japanese DH patients showed fibrillar IgA deposition in the papillary dermis. In contrast to the result in a previous review of Japanese DH, which pointed out a higher frequency of fibrillar IgA deposition than that of granular IgA deposition [59], our study revealed that most common IgA deposition in Japanese DH was the granular pattern. However, the frequency of fibrillar IgA deposition is still high, when compared to that in Caucasian DH [97].

When compared between the granular and fibrillar groups, male patients in the granular group were older than those in fibrillar group although apparent statistical significance was not obtained. The lesions in the fibrillar group seemed to spare the predilection sites of DH, such as elbow, knee, and buttock, while the lesions in the granular group frequently involve these sites.

Recently cases with combined granular and fibrillar IgA deposition were reported although it is not clear whether this combined deposition is just extraordinary or incidental findings or not [17, 19, 41, 49, 71, 79, 81]. Only one case showed cluster IgA deposition although detailed data were not available [80]. The results of IIF studies showed no specific IgA antibodies. This was different from Caucasian results, which showed 63.5% positivity against the endomysium of smooth muscle [92].

 In Caucasian patients, DH has a clear relationship to CD and is considered to be the cutaneous expression of GSE [87, 89]. Although most of Caucasian DH patients have clinically no or mild gastrointestinal symptoms, they are treated with strict GFD [89]. The maintenance of GFD is important because gluten challenge leads to a flare of the cutaneous symptoms in Caucasian DH [88]. However, this is not the case in Japanese DH. Only 2 Japanese DH patients were treated with strict GFD [47, 59], while most patients continued to take a normal diet and were successfully controlled by dapsone or topical steroid. In addition, the lesions of some Japanese DH patients were completely cleared by short-term administration of dapsone or topical steroid while taking a normal diet. Moreover, except for the 3 patients with GSE, no patients developed clinical symptoms of gluten sensitivity throughout the course on a normal diet. Taken together, Japanese DH may not be closely associated with gluten sensitivity, in contrast to Caucasian DH.

The final diagnosis of CD is made by the results of jejunum biopsy, but not by clinical symptoms. In fact, the jejunum biopsy in 2 Japanese DH patients with no clinical symptoms of gluten sensitivity revealed villous atrophy [14, 39]. However, the fact that the jejunum biopsy of 3 patients with no clinical symptoms of gluten sensitivity revealed no pathological changes may raise the possibility that some Japanese DH patients did not have GSE. To confirm the exact association of GSE in Japanese DH, jejunum biopsies are necessary. However, most Japanese DH patients refused jejunum biopsy and GFD because of no clinical symptoms [84]. Accordingly, it may be difficult to clarify the exact association of GSE in Japanese DH based on the histopathological changes of the jejunum. Nevertheless, GSE in Japanese DH seems rare, considering the good response to the dapsone or topical steroid therapy during taking a normal diet. The rarity of GSE in Japanese DH patients is considered to be well correlated with the extreme rarity of CD in Japan [98].

 Regarding genetic testing for Caucasian DH, the absence of HLA-DQ2 or DQ8 has a high negative predictive value because patients lacking these alleles are very unlikely to have DH [87, 99]. However, because the prevalence of these alleles among the Caucasian population is rather high, positive results in the HLA test are not sufficient to diagnose DH. In contrast, our study disclosed that Japanese DH patients never had HLA-DQ2 or -DQ8. Although no frequencies of HLA class I antigens in Japanese DH patients were increased in comparison with the controls among Japanese [90], there was a slightly increased frequency of HLA-DR9 in all the Japanese DH patients examined for HLA. Moreover, patients in the granular group showed a statistically significant frequency of HLA-DR9 when compared to Japanese normal controls. Since the number of data available was small, further analyses are needed to conclude whether Japanese DH is associated with specific HLA alleles.

 Serologic tests of IgA antigliadin and antireticulin antibodies are no longer considered to be sensitive and specific markers of DH [87]. Instead, tests for IgA antibodies to eTG, tTG, and endomysium are considered to be useful diagnostic tools for Caucasian DH [87, 88]. Intestinal damage caused by exposure to gluten was suggested to produce IgA anti-tTG and anti-eTG antibodies [100]. Among them, eTG, rather than tTG is considered as the domain autoantigen in DH [101]. Recent studies reported a high sensitivity and specificity of eTG ELISA for DH [102, 103]. In a few Japanese DH patients who were tested for DH-related autoantibodies, no autoantibodies except for IgA anti-eTG antibodies were detected. A recent report suggested that the absence of GSE in Japanese DH may be due to an absence of anti-tTG antibodies, and that anti-eTG antibodies may be a diagnostic marker for Japanese DH [84]. These serologic tests, particularly for IgA anti-eTG antibodies should be performed in the future studies of Japanese DH.

Caucasian DH is known to be associated with a number of autoimmune conditions, including thyroid disease, type I DM, and autoimmune connective tissue diseases such as Sjögren syndrome, rheumatoid arthritis, and lupus erythematosus [89]. However, in Japanese DH, these diseases were relatively rare. Most Japanese DH patients with DM had type II DM. A higher risk of non-Hodgkin lymphoma was also reported in Caucasian DH [89], but this relation was not found in Japanese DH.

In summary, the absence of HLA-DQ2/DQ8, the inability to identify CD in most cases, the predominance of fibrillar IgA, and the unusual distribution of clinical lesions in Japanese patients suggest that Japanese DH may be a subset of DH patients and have a pathogenesis which is different from that currently proposed in Caucasian DH patients.

## 5. Conclusions

In conclusion, we reported the differences between Caucasian DH and Japanese DH. The characteristics of Japanese DH are (1) a high frequency of fibrillar IgA deposition in the papillary dermis, (2) a rare occurrence of GSE, (3) the absence of HLA-DQ2 or -DQ8, and (4) a rare association with autoimmune diseases or lymphomas. Although a previous Japanese DH review reported the prevalence of fibrillar IgA deposition, our study revealed that Japanese DH patients showed granular IgA deposition more frequently than fibrillar IgA deposition. In addition, we found that HLA-DR9 was frequently detected in Japanese DH, particularly in the granular group. These data suggest distinct characteristics of Japanese DH and raise the possibility that Japanese DH has a different pathogenesis from Caucasian DH. Serological tests for IgA anti-eTG antibodies and HLA genotyping should be performed in the future because Japanese DH may frequently have anti-eTG antibodies and HLA-DR9.

## Figures and Tables

**Table 1:** Clinical characteristics of 91 patients.

	*N* or age/*N* of data available	
Gender		
Male	61/91	
Female	30/91	
Age at the initial visit, mean ± SD (range), years	46.6 ± 19.9/91	(1–87)
Male	51.5 ± 20.5/61	(1–87)
Female	36.8 ± 14.1/30	(18–72)
Age at onset, mean ± SD (range), years	43.8 ± 18.6/75	(1–87)
Male	48.5 ± 19.6/48	(1–87)
Female	35.3 ± 13.0/27	(14–72)
Site of lesion		
Elbow	33/84	(39.3%)
Knee	29/84	(34.5%)
Buttock	30/84	(35.7%)
Elbow and/or knee and/or buttock	37/84	(44.0%)
Face	11/84	(13.1%)
Ear	9/84	(10.7%)
Neck	8/84	(9.5%)
Scalp	6/84	(7.1%)
Groin	4/84	(4.8%)
At least one predilection site	49/84	(58.3%)
Extremities*	41/84	(48.8%)
Trunk**	55/84	(65.5%)
Whole body***	6/84	(7.1%)
IgA deposition in the papillary dermis		
Granular	50/91	(54.9%)
Fibrillar	33/91	(36.3%)
Granular and fibrillar	7/91	(7.7%)
Cluster	1/91	(1.1%)
Other deposition in the papillary dermis		
C3	20/91	(22.0%)
IgG	9/91	(9.9%)
IgM	4/91	(4.4%)
Fibrinogen	2/91	(2.2%)
GSE	3/91	(3.3%)
Jejunum mucosa biopsy		
Villous atrophy****	3/6	(50.0%)
No change*****	3/6	(50.0%)
Associated diseases		
Diabetes mellitus	8/91	(8.8%)
Lymphoma	3/91	(3.3%)
Thyroid disease	1/91	(1.1%)
Sjögren syndrome	1/91	(1.1%)
HLA antigen^§^		
DR4	13/31	(41.9%)^#^
DR9	15/31	(48.4%)^##^
Other antibodies		
Antireticulin antibodies	0/9	(0.0%)
Antigliadin antibodies	0/3	(0.0%)
Antiendomysial antibodies	0/3	(0.0%)
Anti-tTG IgA antibodies	0/2	(0.0%)
Anti-eTG IgA antibodies	2/2	(100.0%)
Therapy		
Dapsone	62/82	(75.6%)
Dapsone + gluten-free diet	7/82	(8.5%)
Gluten-free diet + topical steroid	1/82	(1.2%)
Topical steroid	9/82	(11.0%)
Others^†^	3/82	(3.7%)

*Not including cases limited only to elbow or knee; **not including cases limited only to buttock, neck, or groin; ***not including cases limited to combination of predilection sites; ****including 1 patient with GSE; *****no patients had GSE; GSE: gluten-sensitive enteropathy; tTG: tissue transglutaminase, eTG: epidermal transglutaminase; ^†^including minocycline and topical steroid, salazosulfapyridine, and zinc oxide ointment; ^§^frequency in HLA antigens of control was depicted from [90]. ^#^ *P* = 0.9, ^##^ *P* = 0.007, corrected *P* = 0.07.

**Table 2:** Comparison of granular and fibrillar deposition groups.

	Granular (*N* = 50)		Fibrillar (*N* = 33)		*t*-test	
	*N* or age/*N* of data available		*N* or age/*N* of data available			
Gender						
Male	33/50	(66.0%)	22/33	(66.7%)		
Female	17/50	(34.0%)	11/33	(33.3%)		
Age at the initial visit, mean ± SD (range), years	47.2 ± 20.1/50	(1–86)	43.8 ± 18.0/33	(16–78)	*P *= 0.436	
Male	53.1 ± 19.5/33	(1–86)	45.9 ± 20.2/22	(16–78)	*P *= 0.071	
Female	35.9 ± 15.9/17	(18–72)	39.6 ± 11.4/11	(22–68)	*P *= 0.780	
Age at onset, mean ± SD (range), years	44.4 ± 18.2/42	(1–73)	39.7 ± 16.0/25	(12–74)	*P *= 0.295	
Male	50.3 ± 17.0/26	(1–73)	40.9 ± 19.4/16	(12–74)	*P *= 0.114	
Female	34.9 ± 15.9/16	(14–72)	37.7 ± 5.7/9	(29–50)	*P *= 0.630	
Site of lesion						
Elbow	23/46	(50.0%)	5/30	(16.7%)		
Knee	20/46	(43.5%)	5/30	(16.7%)		
Buttock	25/46	(54.3%)	4/30	(13.3%)		
Elbow and/or knee and/or buttock	30/46	(65.2%)	7/30	(23.3%)		
Face	8/46	(17.4%)	3/30	(10.0%)		
Ear	7/46	(15.2%)	2/30	(6.7%)		
Neck	3/46	(6.5%)	5/30	(16.7%)		
Scalp	5/46	(10.9%)	1/30	(3.3%)		
Groin	4/46	(8.7%)	0/30	(0.0%)		
At least one predilection site	36/46	(78.3%)	12/30	(40.0%)		
Extremities*	20/46	(43.5%)	17/30	(56.7%)		
Trunk**	27/46	(58.7%)	22/30	(73.3%)		
Whole body***	3/46	(6.5%)	3/30	(10.0%)		
Other deposition in the papillary dermis						
C3	13/50	(26.0%)	4/33	(12.1%)		
IgG	5/50	(10.0%)	4/33	(12.1%)		
IgM	3/50	(6.0%)	1/33	(3.0%)		
Fibrinogen	2/50	(4.0%)	0/33	(0.0%)		
Small bowel disease	3/50	(6.0%)	1/33	(3.0%)		
Associated diseases						
Diabetes mellitus	5/50	(10.0%)	2/33	(6.1%)		
Lymphoma	3/50	(6.0%)	0/33	(0.0%)		
Thyroid disease	0/50	(0.0%)	1/33	(3.0%)		
Sjögren syndrome	1/50	(2.0%)	0/33	(0.0%)		
HLA antigen^§^						
DR4	4/14	(28.6%)^#^	8/13	(61.5%)^##^		
DR9	9/14	(64.3%)^¶^	3/13	(23.1%)^¶¶^		

*Not including cases limited only to elbow or knee; **not including cases limited only to buttock, neck, or groin; ***not including cases limited to combination of predilection sites; ^§^frequency in HLA antigens of control was depicted from [90]. ^#^ *P* = 0.3 (versus controls), *P* = 0.09 (versus Fibrillar); ^##^ *P* = 0.2 (versus controls), ^¶^ *P* = 0.002 (versus controls, corrected *P* = 0.02), *P* = 0.03 (versus Fibrillar), ^¶¶^ *P* = 0.8 (versus controls).
